# Targeting the 8-oxodG Base Excision Repair Pathway for Cancer Therapy

**DOI:** 10.3390/cells14020112

**Published:** 2025-01-14

**Authors:** Anna Piscone, Francesca Gorini, Susanna Ambrosio, Anna Noviello, Giovanni Scala, Barbara Majello, Stefano Amente

**Affiliations:** 1 Department of Molecular Medicine and Medical Biotechnologies, University of Naples ‘Federico II’, 80131 Naples, Italy; 2 Department of Biology, University of Naples ‘Federico II’, 80138 Naples, Italy

**Keywords:** BER, 8-oxodG, cancer therapy

## Abstract

Genomic integrity is critical for cellular homeostasis, preventing the accumulation of mutations that can drive diseases such as cancer. Among the mechanisms safeguarding genomic stability, the Base Excision Repair (BER) pathway plays a pivotal role in counteracting oxidative DNA damage caused by reactive oxygen species. Central to this pathway are enzymes like 8-oxoguanine glycosylase 1 (OGG1), which recognize and excise 8-oxo-7,8-dihydro-2′-deoxyguanosine (8-oxodG) lesions, thereby initiating a series of repair processes that restore DNA integrity. BER inhibitors have recently been identified as a promising approach in cancer therapy, increasing the sensitivity of cancer cells to radiotherapy and chemotherapy. By exploiting tumor-specific DNA repair dependencies and synthetic lethal interactions, these inhibitors could be used to selectively target cancer cells while sparing normal cells. This review provides a robust reference for scientific researchers, offering an updated perspective on small-molecule inhibitors targeting the 8-oxodG-BER pathway and highlighting their potential role in expanding cancer treatment strategies.

## 1. Introduction

Maintaining genomic integrity is essential for cellular homeostasis, as it prevents the accumulation of mutations that could lead to various diseases, including cancer. This integrity is safeguarded by a complex network of DNA repair pathways. The dysregulation of these pathways contributes to increased genomic instability and tumor progression [1,2,3]. Genomic instability promotes tumor development by causing chromatin disorganization, mutations, and altered gene regulation [4,5]. Among the mechanisms preserving genomic stability, the Base Excision Repair (BER) pathway is pivotal in counteracting oxidative DNA damage. This damage is primarily caused by reactive oxygen species (ROS), which can induce lesions such as 8-oxo-7,8-dihydro-2′-deoxyguanosine (8-oxodG), one of the most prevalent forms of oxidative DNA damage.

**Figure 1:**
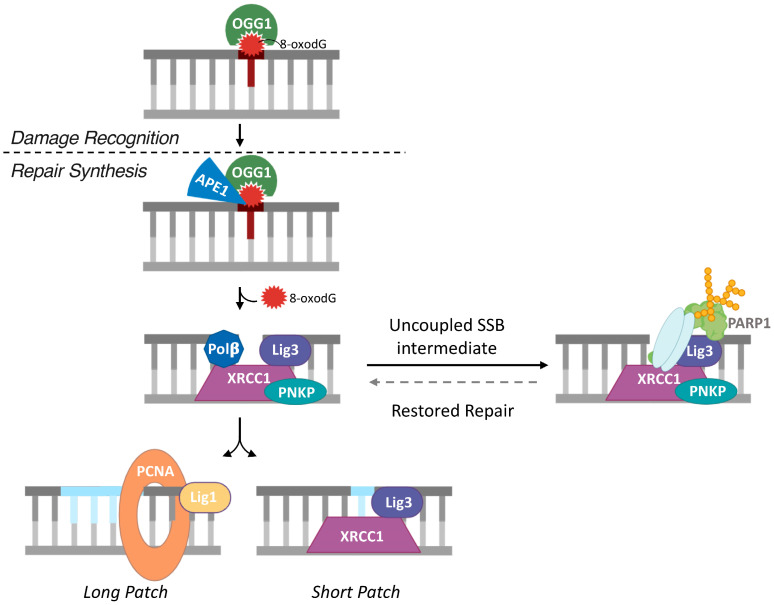
Scheme of 8-oxodG-BER pathway: upon detecting 8-oxodG, OGG1 removes the damaged base and the APE1 endonuclease processes the resulting AP site, generating a single-strand break (SSB) intermediate. If this intermediate is not immediately repaired, PARP1 may recognize and bind to the SSB. However, PARP1 is not essential for accurate repair if the BER pathway is functioning properly. The BER pathway is completed when the DNA polymerase β (POL β) incorporates a single nucleotide, and DNA Ligase 3 (LIG3), along with the scaffold protein XRCC1, seals the nick to complete the repair. If the BER machinery fails to complete ligation, long patch repair is thought to take over.

The BER pathway identifies and repairs 8-oxodG lesions (hereafter named 8-oxodG-BER) and involves a series of highly conserved proteins [6]. In particular, the 8-oxodG-BER process can be divided into two main stages—damage recognition and repair synthesis—as illustrated in Figure 1, which outlines the sequential steps and enzymes involved. The first stage, damage recognition, is mediated by the enzyme 8-oxoguanine glycosylase 1 (OGG1). OGG1 specifically identifies and excises 8-oxodGs by cleaving the corresponding N-glycosidic bond and generating an abasic site. This step is critical for preventing mutations and maintaining genomic stability [7,8].

Interestingly, recent studies characterized OGG1 as an “epigenetic reader” that responds to oxidative stress by relocating itself to regions of open chromatin in coordination with other BER proteins [9,10]. In addition to OGG1, human homologs of NEIL glycosylases can recognize 8-oxodG, particularly in single-stranded DNA regions [11,12,13]. Both OGG1 and NEIL glycosylases are equipped with AP lyase activity, allowing them to cleave the DNA backbone at the 3′ position adjacent to an abasic site through a β-elimination reaction, forming a 3′-phospho-α, β-unsaturated aldehyde (PUA) [14,15].

The subsequent stage, the repair synthesis step, is a dynamic process that converts abasic sites into single-strand breaks (SSBs) (Figure 1). Once the damaged base is excised, enzymes such as polynucleotide kinase–phosphatase (PNKP) or AP endonuclease 1 (APE1) process the abasic sites by removing blocking groups like 3′-PUA or 3′-phosphate (3′-PO4), thus generating SSBs, while BER intermediates produce 3′-OH ends suitable for DNA synthesis [6].

In short-patch BER (Figure 1), DNA polymerase β (POL β) incorporates a single nucleotide and DNA Ligase 3 (LIG3), along with the scaffold protein XRCC1, seals the nick to complete the repair process [6,16,17]. For long-patch BER (Figure 1), DNA polymerase δ performs strand displacement synthesis, with the Proliferating Cell Nuclear Antigen (PCNA) acting as a sliding clamp loaded via replication factor C (RFC). The displaced flap is then cleaved by Flap Endonuclease 1 (FEN1) and DNA Ligase I finalizes the repair [18,19,20].

Another protein that may play a role in 8-oxodG-BER is Poly(ADP-ribose) polymerase (PARP) and, in particular, the extensively studied PARP1 isoform. PARP1 is primarily involved in repairing DNA SSBs and is not required for effective BER in vitro biochemical assays [21], indicating it does not play a direct role in BER. Instead, PARP1 binds to SSB intermediates that become uncoupled during the BER process (Figure 1). Upon binding, PARP1 catalyzes the addition of ADP-ribose units to target proteins, such as histones, through a modification known as PARylation. This modification promotes the recruitment of other DNA repair proteins, such as XRCC1 [22], which are essential for completing the BER process and ensuring damaged DNA is repaired.

## 2. Targeting the 8-oxodG-BER Proteins

The disruption or inhibition of the BER pathway in cancer cells has become an area of growing interest in cancer research and therapy. BER inhibitors have shown significant potential in increasing the sensitivity of cancer cells to DNA-damaging treatments, such as radiotherapy and chemotherapy. These inhibitors can enhance the effectiveness of such treatments, particularly in tumors, where the overexpression of BER proteins allows for cancer cells to resist standard therapies [23].

Radiotherapy and chemotherapy induce a wide spectrum of DNA lesions within tumor cells, including base damage, single-strand breaks (SSBs), and, indirectly, double-strand breaks (DSBs) through replication stress [24]. The increased activity of BER repairs this damage, enabling tumor cells to survive and persist despite treatment [25]. Therefore, combining BER inhibitors with these conventional therapies could enhance their DNA damage potential, overwhelming the cancer cells’ repair mechanisms and promoting cell death.

An alternative strategy involves the use of BER inhibitors to treat tumors that exhibit deficiencies in other DNA repair pathways. Tumor cells often compensate for defective repair mechanisms by relying on an alternative repair process. By selectively inhibiting these compensatory repair pathways, it becomes feasible to target tumor cells while sparing normal cells, which retain functionality in the repair pathway that is compromised in cancer cells [26]. This strategy hinges on the concept of synthetic lethality, wherein the simultaneous disruption of two genetic or molecular mechanisms leads to cell death, offering a targeted avenue for therapeutic intervention.

Moreover, BER inhibitors can heighten the sensitivity of cancer cells to intrinsic stressors, such as oxidative stress, by amplifying DNA damage to levels that exceed the cell’s repair capacity [27]. This tactic capitalizes on the elevated oxidative stress commonly observed in cancer cells, making them particularly susceptible to the disruption of DNA repair machinery. By exploiting this inherent vulnerability, BER inhibitors hold promise for enhancing the precision and efficacy of cancer therapies.

At present, only PARP1 inhibitors are clinically approved to target BER-related pathways. However, ongoing research has identified several small-molecule inhibitors with potential therapeutic benefits.

In recent years, a growing number of small molecules have been developed to target the activity of BER proteins. This review focuses on the proteins within the BER pathway specifically involved in repairing 8-oxodG lesions, highlighting small molecules designed to inhibit their activity (Table 1).

### 2.1. OGG1 Inhibitors and Activators

Growing evidence suggests that inhibiting OGG1 may be useful in the treatment of certain cancers. However, drugs designed specifically to target OGG1 have yet to reach clinical trials. In the first high-throughput screening for human OGG1 inhibitors, a series of promising compounds were identified. Among these, O151, SU0268 and SU0383 emerged as the most effective inhibitors of OGG1 glycosylase activity (Table 1) [28,29,30]. In addition, a small molecule with anti-inflammatory properties, TH5487, was developed to inhibit OGG1 (Table 1). TH5487 inhibits OGG1 from binding to and repairing 8-oxodG, leading to the inhibition of proinflammatory pathway genes (Table 1) [25]. Moreover, Tanner et al. demonstrated that, in fibroblast cells, TH5487 reduces the expression of pro-fibrotic genes and myofibroblast transition. In a mouse model of bleomycin-induced pulmonary fibrosis using male C57BL6/J mice, TH5487 specifically reduces lung remodeling, inflammatory cell infiltration, and proinflammatory mediator levels [10]. The OGG1 competitive inhibitors TH5487 and SU0268 were shown to have great promise and are considered potential novel strategies for the treatment of inflammatory illnesses and cancer therapy [27,31]. In addition to their interesting potential in therapeutic applications, OGG1 inhibitors serves as valuable tools for basic research, providing significant insights into the molecular mechanisms of OGG1 in transcriptional regulation and DNA repair. Two off-target effects of OGG1 competitive inhibitors, TH5487 and SU0268, on mitotic progression and efflux pumps were revealed by Tanushi et al.; the observed effects are independent of OGG1 activity [32]. Consequently, it is crucial to further investigate the chemical mechanisms of these molecules’ actions and develop more specialized, optimized molecules. Notably, OGG1, by counteracting 8-oxodG, facilitates NF-κB binding to DNA targets before the removal of 8-oxodG, thereby promoting cancer progression through several mechanisms, such as alterations in the vascular network, the expression and secretion of molecules that modulate innate immunity, and phenotypic transitions between cancer and stromal cells [9]. According to the current research, OGG1’s ability to facilitate the binding of NFκB and Myc to their respective DNA motifs can change cancer progression and make cancer cells resistant to chemotherapy and radiation [9]. Consequently, as suggested in recent research [33], small compounds suppressing the production of the epigenetic-like mark 8-oxodG and/or the recognition of its genomic substrate by OGG1 may have clinical utility. Gaining more insight into the ways in which ROS, OGG1, NFκB, and Myc interact could be useful in the comprehension and eventual treatment of malignant diseases.

In addition to OGG1 inhibitors, increasing attention has been directed toward activators that significantly enhance the basal catalytic activity of OGG1. These hold promise in counteracting the harmful effects of environmental oxidants and mutagens, particularly in addressing specific human OGG1 gene mutations. OGG1’s catalytic core is in its C-terminal domain, where key residues enable base excision and lyase activities [34]. Lys249 acts as a nucleophile, forming a covalent intermediate with the C1’ of the damaged nucleotide, enabling the β-elimination reaction, while Asp268 and His270 are involved in proton transfer and cleaving the DNA backbone during β,δ-lyase activities. Phe319 aids in recognizing and stacking with 8-oxodG to ensure specificity. Additionally, Ser326 plays a critical role in positioning the DNA for efficient base excision. Mutations like Ser326Cys can reduce OGG1’s repair efficiency, leading to genomic instability and an increased risk of cancer [35]. For example, studies have demonstrated that cells expressing the hOGG1S326C variant exhibit a lack of 8-oxodG cleavage activity in mitochondrial extracts. However, small-molecule activators of OGG1 were found to enhance mitochondrial DNA (mtDNA) repair, providing protection against the disease-associated Ser326Cys variant of hOGG1 [35].

Recent research has identified other small-molecule activators, including TH12117 and TH10785 (Table 1), which significantly enhance OGG1 activity. TH10785 interacts with phenylalanine-319 and glycine-42 residues within OGG1, boosting its enzymatic activity and revealing a novel catalytic function as a β,δ-lyase. This shift reorients OGG1’s activity toward reliance on polynucleotide kinase phosphatase (PNKP1), enabling the recognition of AP sites. As a result, it facilitates the repair of oxidative DNA damage while opening new repair pathways with therapeutic relevance for diseases and aging processes. Similarly, TH12117 interacts with the same amino acid residues, substituting excised bases and initiating β-elimination events. This process releases the Lys249 residue, thereby enabling OGG1 to efficiently restart its catalytic cycle. The use of these two OGG1 activators shifts the 8-oxodG-BER pathway from dependency on APE1 to dependency on PNKP1. This transition facilitates a targeted overload of a novel repair pathway, dealing with lesions arising from both 8-oxodG and AP sites [36,37].

Further research has led to the development of hybrid molecules, such as TH12161, which are derived from previous activators and continue to function as OGG1 activators [31,38].

These findings underscore the potential of these compounds to have wider therapeutic applications, particularly in the context of oxidative DNA damage and repair.

### 2.2. APE1 Inhibitors

APE1 is also being investigated as a target for cancer therapy. APE1 has been described as both a BER endonuclease and a redox hub for several transcription factors that are involved in a wide range of cellular processes, including growth, inflammation, and angiogenesis [39,40]. The inhibition of both endonuclease and redox activities can be useful in cancer treatment. Inhibiting the DNA-repair function of APE1 may increase the susceptibility of drug-resistant tumors to DNA-damaging drugs, leading to increased levels of apoptosis. In addition, inhibiting the redox function of APE1 would prevent transcription factors from binding to DNA, thereby altering the expression of target genes [39]. One of the first molecules investigated for its effect on APE1 repair activity was methoxyamine (MX) (Table 1). MX acts as an indirect inhibitor of the repairing function of APE1 [41] by binding sugar aldehydes at AP sites. As a result, the AP endonuclease is unable to recognize the site of damage and the DNA remains unrepaired. In pre-clinical models, methoxyamine has been shown to improve the efficacy of DNA-damaging drugs such as temozolomide, pemetrexed, fludarabine, and 5-fluorouracil by significantly reducing tumor growth. Phase I trials investigated the safety and tolerability of combining pemetrexed with methoxyamine (NCT00692159) and fludarabine with methoxyamine (NCT01658319) and found both combinations to be safe and well-tolerated [42]. A phase II trial was conducted to evaluate the combination of methoxyamine and temozolomide in patients with recurrent glioblastoma (NCT02395692). However, the study did not meet the pre-specified response criteria required for progression to the next phase. Another phase I/II trial investigating the combination of methoxyamine and temozolomide in patients with relapsed solid tumors and lymphomas was completed, but the results have not yet been reported (NCT01851369) [39].

Several other small-molecule inhibitors have been designed to directly target APE1 catalytic activity, as reviewed by [40]. When combined with chemotherapeutics such as methyl methanesulfonate (MMS) and temozolomide (TMZ), these inhibitors showed increased sensitivity in various tumor cell lines. However, to date, none of these inhibitors has undergone extensive in vivo testing or entered clinical trials [40].

Significant progress has been made with APX3330 (Table 1), a small-molecule inhibitor that specifically targets the redox function of APE1 in various cancer models, including prostate cancer, colon cancer, and pancreatic cancer [43,44,45,46,47]. It demonstrated its effectiveness in reducing the growth of tumors, lowering cell proliferation, and suppressing transcriptional activity. Following a successful phase I clinical trial in 2017 (NCT03375086), APX3330 entered a phase II trial for diabetic retinopathy and macular edema in 2020, demonstrating its safety and tolerability [40,48]. A second generation of compounds targeting APE1, such as APX2007 and APX2014 (Table 1), as well as new analogs like RN8-51 (Table 1), have been created, indicating potential for their further development as anti-cancer agents [49,50]. Among these, APX2009 showed neuroprotective properties against cisplatin- and oxaliplatin-induced toxicity, without compromising the antitumour activity of platins, as well as potent tumor cell-killing activity [43].

Dietary and natural compounds like soy isoflavones, resveratrol, and curcumin also demonstrate the ability to inhibit APE1 redox function [40]. In several human cancer models, these compounds markedly reduce both APE1 and NF-κB protein activity, enhancing the cytotoxic effects of DNA-damaging therapies [40].

A few molecules have been demonstrated to inhibit both APE1 endonuclease and redox function. These include gossypol (Table 1), a natural polyphenolic aldehyde known to directly interact with APE1 [51]. A recent clinical trial (NCT00540722) was designed to explore the potential clinical benefits of combining gossypol with docetaxel and cisplatin in patients with non-small cell lung cancer with elevated APE1 expression. The trials showed improved outcomes in terms of prolonged progression-free survival and overall survival in patients treated with gossypol, although statistical significance has not yet been fully established [52]. A compound derived from gossypol, AT-101 (Table 1), was shown to have significant anti-cancer activity, but its mechanism of sensitizing cancer cells to chemotherapy requires further study [53].

### 2.3. PARP Inhibitors

PARP inhibitors (PARPi) represent a class of anti-cancer agents that function by competing with nicotinamide adenine dinucleotide (NAD+) to bind to the catalytic site of PARP enzymes.

Although the precise mechanisms behind the anti-cancer effects of PARP inhibitors remain incompletely defined, recent research has provided valuable insights into their mode of action, although a definitive consensus has yet to be established. By inhibiting PARP, these agents disrupt DNA repair and cause SSB accumulation. During DNA replication or cell division, unresolved SSBs can escalate into more deleterious DSBs, thereby compromising genomic stability. Another proposed mechanism is PARP trapping, where PARP inhibitors prevent the release of PARP enzymes from DNA, resulting in the formation of PARP-DNA adducts. These trapped complexes block the recruitment of DNA-repair proteins, and interfere with normal nuclear functions, further increasing the formation of DSBs and enhancing the cytotoxic effects of PARP inhibition. These DSBs can be repaired by HR in cells with functional homologous recombination repair (HRR) pathways. However, in HRR-deficient cells, the inability to repair these breaks leads to genomic instability and cell death. In cells whose DNA repair mechanisms are already compromised, such as those with BRCA1 or BRCA2 mutations, which are common causes of HRR pathway defects, these DSBs are particularly damaging. In such cases, the inhibition of PARP activity induces a “synthetic lethality” effect, where the combination of PARPi and defective HRR leads to lethal consequences for the cell. PARPi have been demonstrated to be effective against tumors that are deficient in HRR through a synthetically lethal interaction [54]. PARPi target cancer cells that are deficient in HRR while sparing normal cells that can still use HRR to repair DNA. Although this effect is particularly pronounced in cancers with BRCA mutations or other HRR pathway defects, the relatively low incidence of BRCA1/2 mutations limits the applicability of PARPi. This limits their use to the treatment of 10–15% of breast and ovarian cancers, 4–7% of pancreatic cancers, and 1.5% of prostate cancers. However, recent research suggests that PARPi may have a wider range of potential applications, including in the treatment of cancers with an alternative HR deficiency or mutations in other DNA damage response genes. In addition, tumors characterized by increased oxidative and replicative stress may be sensitive to PARPi [54]. Several PARPi are currently available. They include olaparib, talazoparib, niraparib, rucaparib, and veliparib (Table 1) [55]. The FDA has approved these inhibitors for cancer therapy, and they have shown significant efficacy in the treatment of cancer [56].

Despite the clinical benefit of PARPi for patients with BRCA1/2 mutations, the emergence of drug resistance continues to be a therapeutic challenge. Although the mechanisms underlying innate resistance remain largely unclear, various acquired resistance mechanisms have been identified. One of the main mechanisms driving resistance to PARPi is the restoration of HRR [57,58,59]. This can occur through secondary reversion mutations or the loss of promoter methylation in HR-related genes, which reinstates the HRR capacity of cancer cells, thereby reducing the efficacy of PARPi [57,58]. Additionally, splice variants such as BRCA1-Δ11q retain partial HRR functionality, further diminishing the effectiveness of PARPi [59]. Resistance can also arise through BRCA1/2-independent mechanisms [60,61]. For instance, the loss of 53BP1 or components of the Shieldin complex facilitates DNA end resection, enabling HRR in BRCA1-deficient tumors [60,61]. When NHEJ factors are deficient, cells may instead rely on theta-mediated end-joining (TMEJ), a less precise repair pathway mediated by DNA polymerase theta (POL θ). BRCA1/2 protects stalled replication forks from nucleases like MRE11; resistance emerges when replication fork degradation is prevented through alternative mechanisms [62]. Mutations in PARP1 or reduced PARG activity diminish PARP1 trapping, undermining the effectiveness of PARPi [63,64]. Lastly, the overexpression of ABCB1 enhances drug efflux, decreasing intracellular PARPi concentrations [65] (for further details, please refer to [66]). However, a deeper understanding of the mechanisms underlying resistance to PARPi is essential for the improvement of these drugs or the optimization of their use in combination with other treatments. Although modern PARPi have been developed to specifically target cancer cells, there is a possibility that they may affect other bodily functions and thus lead to undesirable side effects. This shows the importance of conducting further research in order to develop more precise therapies that reduce these unwanted effects. The combination of PARPi with other treatments, such as chemotherapy, radiation therapy, or immunotherapy, shows considerable promise, particularly in terms of enhancing the overall effectiveness of PARPi when treating cancers that are difficult to treat with conventional methods [67]. The FDA has recently approved combination therapies involving niraparib with abiraterone, olaparib with abiraterone, and talazoparib with enzalutamide in metastatic castration-sensitive prostate cancer [68].

### 2.4. DNA Polymerase β Inhibitors

DNA polymerase beta (POL β) is a key enzyme required for BER. Its role makes it a promising target for increasing tumor sensitivity to DNA-damaging agents. The inhibition of POL β leads to the accumulation of apurinic acid/apyrimidinic acid (AP) sites. If left unrepaired, these sites lead to the accumulation of SSBs, which stall the DNA replication fork during the S phase, leading to the formation of double-stranded breaks [69]. Several small-molecule inhibitors of POL β have been developed. NSC666715 is a small-molecule inhibitor of POL β that inhibits the strand displacement activity of POL β in LP-BER (Table 1). This inhibition induces cell cycle arrest in the S phase and triggers senescence and apoptosis in colorectal cancer cells. In addition, NSC666715 potentiates the effect of TMZ to induce cell senescence in these cell lines [70].

Pro-13 is an irreversible inhibitor of POL β (and POL λ). It exhibits weak cytotoxicity against human cervical carcinoma (HeLa) cells, but its effect is enhanced when combined with methyl methanesulfonate (MMS). The cytotoxic effects are maintained even after MMS is removed. In the presence of Pro-13, DNA from MMS-treated HeLa cells undergoes a threefold increase in abasic sites due to the inhibition of DNA repair processes (Table 1). Notably, Pro-13 enhances the cytotoxicity of MMS more than any other inhibitor of BER enzymes reported to date [71].

Natamycin, an antibiotic and antifungal agent, has been shown to inhibit the strand displacement activity of POL β at concentrations of 2–5 nM (Table 1). At higher concentrations (in the μM range), natamycin inhibits both POL β and LIGI. Through BER inhibition, natamycin has been shown to suppress the proliferation of prostate cancer cells [71]. In both the short-patch and long-patch BER sub pathways [72,73], the DNA polymerase POL B is essential for cellular resistance against a range of chemotherapeutic drugs and radiation [74]. Oleanolic acid (OA), in combination with 6-thioguanine (6-TG), induces lethal DNA damage, leading to apoptosis in mismatch repair (MMR)-deficient cells. This combination has been shown to have synergistic effects in vitro and to significantly lower leukemic burden in xenograft models of MMR-deficient acute lymphoblastic leukemia [75]. Additionally, the inhibitor Aphidicolin targets POL δ/ε and POL α, specifically inhibiting long-patch BER and sensitizing chronic lymphocytic leukemia cells to purine analogs, suggesting a potential therapeutic role in enhancing the apoptotic response in CLL patients [76].

### 2.5. Lig/XRCC1 Inhibitors

Cancer research is currently also focused on developing ligase inhibitors that can selectively target the different isoforms of ligases. This targeted strategy is promising in cancer therapy as ligase inhibitors have the potential to enhance the effects of DNA-damaging agents. The expectation is that these inhibitors could be able to preserve the DNA damage induced by such agents by disrupting the ligation process, which is the final step in DNA repair, where damaged DNA strands are rejoined. Consequently, these inhibitors have the potential to increase the effectiveness of treatments that cause DNA breaks, such as chemotherapy or radiation, by preventing the repair of such lesions [77]. In the context of ligase IV inhibition, SCR7 has been shown to cause the accumulation of DNA DBSs, leading to apoptosis in cancer cells. Furthermore, SCR7 inhibited nick ligation through ligase IIIa/XRCC1. Given the role of ligase IIIa/XRCC1 in BER, further research is required to investigate the effects of SCR7 on the BER pathway (Table 1) [78]. In addition, several compounds with specificity for LIG I, LIG II, and LIG IIIα, or all DNA ligases, were developed based on the molecular structure of hLIG I complexed with nicked DNA. For example, L82 inhibits human DNA ligase I and has cytostatic properties (Table 1). L67 exhibits activity against LIG I and LIG III, while L189 is active against hLIG I, hLIG III, and LIG IV (Table 1). Interestingly, L67 and L189 demonstrate cytotoxicity in MCF7, HeLa, and HCT116 cells and were found to increase the sensitivity of MCF7 breast cancer cell lines to MMS or ionizing radiation, whereas they had no effect on the sensitization of normal breast cell lines [79].

## 3. Therapeutic Targeting of the 8-oxodG-BER Pathway: Future Perspectives and Clinical Implications

The 8-oxodG-BER pathway has emerged as a promising target for cancer therapy due to its critical role in maintaining genomic stability. The dysregulation of BER proteins often observed in numerous cancer types offers a unique vulnerability that can be exploited therapeutically, particularly in tumors with high oxidative stress.

Among the proteins involved in the BER pathway, those associated with the repair synthesis phase, such as XRCC1, LIG3, and POL β, are particularly attractive therapeutic targets. Unlike glycosylases, repair synthesis proteins lack functional redundancy, meaning their inhibition can lead to the accumulation of irreparable SSBs. These SSBs, under replication stress, escalate into DSBs, driving cancer cells toward cell death due to their severe genomic instability [80,81]. By disrupting the repair synthesis phase, these inhibitors increase the accumulation of DNA lesions in cancer cells, selectively impairing their survival while sparing normal cells, which are less reliant on BER due to their lower oxidative stress levels.

In addition, an exciting avenue for improving the therapeutic outcomes of 8-oxodG-BER-targeting strategies is their combination with other treatment modalities, such as radiotherapy, chemotherapy, and immune checkpoint inhibitors. By combining BER inhibitors with conventional DNA-damaging therapies, the repair capacity of cancer cells can be overwhelmed, leading to enhanced cell death. Indeed, inhibitors targeting POL β or XRCC1 demonstrated the ability to potentiate the effects of DNA-damaging treatments such as radiotherapy and chemotherapy [82,83]. This combination could be particularly beneficial in tumors that are resistant to standard treatments due to their efficient DNA repair mechanisms.

**Figure 2:**
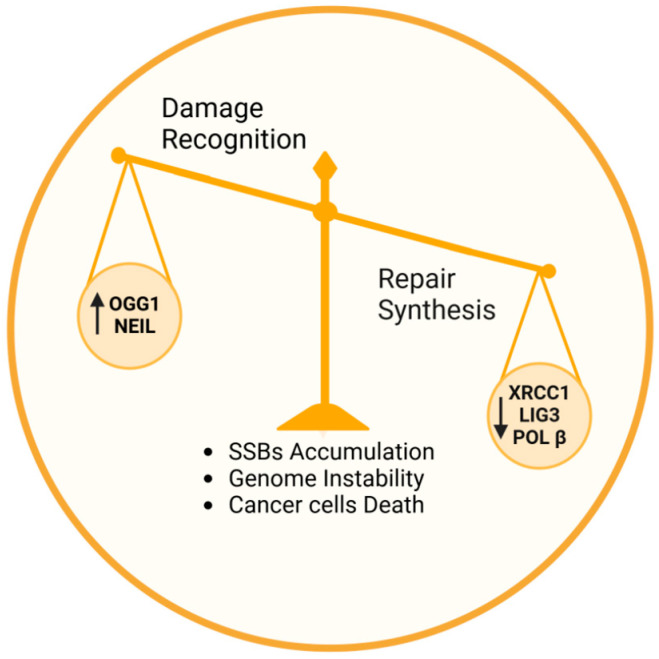
Schematic representation of the BER pathway, visualized as a balanced framework: damage recognition via DNA glycosylases like OGG1 complements repair synthesis and ligation driven by XRCC1, LIG 3, and POL β. Maintaining balance between these forces is crucial for genome stability, while targeted manipulation, by activating (arrow pointing up) damage recognition and inhibiting (arrow pointing down) the repair synthesis, offers a novel strategy to induce cancer cell death through genomic instability.

Furthermore, the 8-oxodG BER pathway is a framework in which damage recognition and repair synthesis represent complementary forces (Figure 2). The balanced interplay between these forces is crucial for maintaining genome stability, and deficiencies in either component can lead to increased mutagenesis, genomic instability, or cell death [80,81,84].

Notably, manipulating the balance within the framework of the 8-oxodG repair pathway could be a promising and as-yet uninvestigated strategy to improve cancer therapies. Indeed, leveraging small-molecule activators, such as those developed for OGG1, opens a new dimension of this therapeutic strategy, which may involve the simultaneous activation of OGG1 and inhibition of repair synthesis proteins. Supported by the promising results obtained from preclinical and clinical studies that explored single molecules targeting individual steps of the BER pathway [33,37,40,42], this innovative dual-action approach would exploit the enhanced formation of SSBs by OGG1 activators, combined with the suppression of XRCC1 or LIG3 or POL β activity, to prevent the resolution of these breaks. Similarly to the synthetic lethality observed with PARP inhibitors, the accumulation of unrepaired SSBs and their subsequent progression to DSBs would overwhelm the repair capacity of defective HRR cancer cells, leading to cell death through genomic instability (Figure 2). Nonetheless, rigorous experimental studies are necessary to validate this innovative therapeutic concept.

The 8-oxodG-BER pathway represents a compelling target for cancer therapy, especially in tumors that experience heightened oxidative stress. Targeting key proteins within this pathway, such as those involved in damage recognition (e.g., OGG1) and repair synthesis (e.g., POL β, XRCC1, LIG3), offers a strategic approach to selectively impair cancer cell survival. The ability to exploit the unique vulnerabilities of cancer cells, which often rely heavily on BER to manage oxidative damage, could significantly enhance the efficacy of existing therapies.

However, despite the promising results from preclinical studies, the clinical application of BER-targeting strategies still faces several challenges. The main hurdle lies in achieving the selective targeting of BER proteins without causing excessive toxicity to normal cells. Since the BER pathway is crucial for maintaining genome integrity in both normal and cancer cells, therapeutic strategies must be designed to selectively exploit the differences in oxidative stress levels and the reliance on the repair pathway between cancerous and healthy tissues. To this end, the identification of reliable biomarkers to predict tumor responsiveness to BER modulation is critical. Such biomarkers could guide patient selection, ensuring that only those most likely to benefit from these therapies are treated.

## 4. Conclusions

In summary, the 8-oxodG-BER pathway represents a promising target for cancer therapy, with the potential to overcome the limitations of current treatment modalities. The development of small-molecule inhibitors and activators, as well as the combination of BER-targeting strategies with other therapeutic approaches, could lead to more effective, personalized cancer treatments. However, further research is required to better understand the mechanisms governing BER and its role in genomic stability, as well as to optimize the therapeutic application of BER modulators. Future clinical trials will be essential in determining the safety, efficacy, and optimal use of these promising therapies in the fight against cancer and other diseases driven by oxidative DNA damage.

## Figures and Tables

**Table 1:** Overview of BER inhibitors in clinical development for the treatment of cancer.

Stage of BER Pathway	Inhibitor	Target/Action	Applications
*Recognition of Damage* 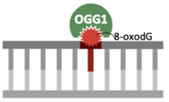	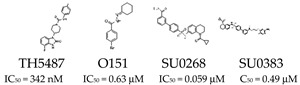	Inhibits OGG1 binding to 8-oxodG or its enzymatic activity.	Used for inflammatory diseases, fibrosis, and research into oxidative stress mechanisms.
	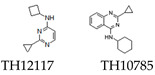	Enhances OGG1 activity, shifting dependency to PNKP1 from APE1.	Explored for therapeutic uses in aging and DNA repair enhancement.
*APE1 Cleavage of AP Site* 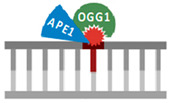	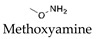	Blocks aldehyde sugar recognition at AP sites, preventing APE1 activity.	Enhances chemotherapy efficacy (e.g., TMZ) in cancers; studied in clinical trials (NCTO0692159).
	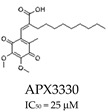	Inhibits the redox function of APE1, suppressing tumor growth and transcriptional activity.	Clinical trials in diabetic retinopathy; preclinical cancer studies (NCT03375086).
	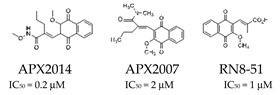	These new analogs target APE1 function, with APX2009 showing neuroprotective properties against cisplatin-and oxaliplatin-induced toxicity without compromising the antitumor activity of platins.	Potential for furth further development as anti-cancer agents, with neuroprotective applications in chemotherapy.
	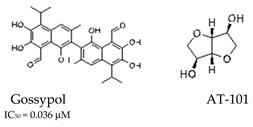	Targets APE1 endonuclease and redox functions, reducing DNA repair and transcription factor activity.	Synergy with chemotherapeutics was demonstrated in models of lung and pancreatic cancer.
*DNA Synthesis* *(POL β)* 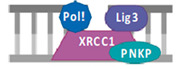	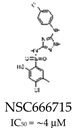	Inhibits strand displacement activity of POLβ; induces cell cycle arrest.	Potentiates chemotherapy effects (e.g., TMZ); studied in colorectal cancer.
	PRO-13 IC_50_ = 0.4 μM	Irreversibly inhibits POLβ; increases AP sites in alkylation-damaged cells.	Sensitizes cancer cells alkylating agents like MMS.
	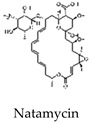	Inhibits POLβstrand displacement and Ligase I activity, impairing BER.	Suppresses cancer cell proliferation, particularly in prostate cancer.
	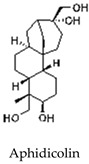	Inhibits POLδ/ε and POLα, impacting long-patch BER.	Studied in chronic lymphocytic leukemia (CLL) and alkylation repair pathways.
*Ligation (Ligase 1/3)* 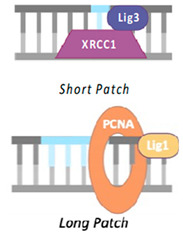	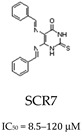	Inhibits LIG IV; disrupts XRCC1-LIG 3 complex; leads to DSB accumulation.	Promotes tumor cell apoptosis; studied in BER and NHEJ pathways.
	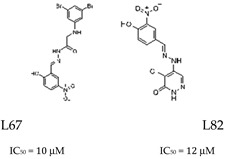	Inhibit LIG l and/or LIG IIIα; impair ligation step in BER.	Cytostatic effects in tumor models; selectively affects cancer cells.
*PARP Activation* 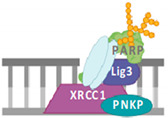	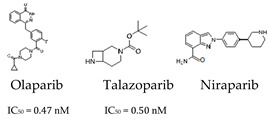	Block ADP-ribosylation, halting DNA repair and trapping PARP-DNA complexes.	Approved for HRR-deficient tumors, including ovarian and prostate cancers.

## Data Availability

Not applicable.
